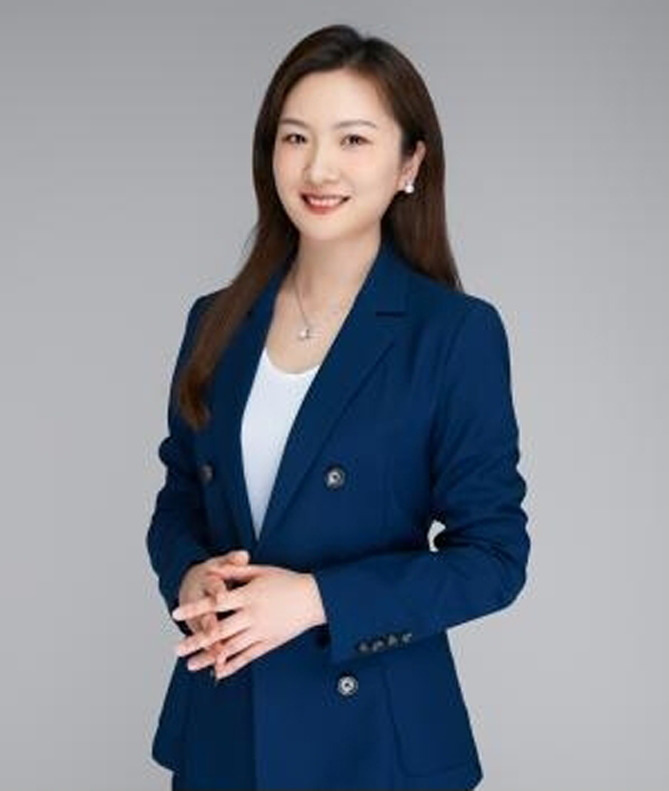# Light People: Elizabeth Rogan

**DOI:** 10.1038/s41377-021-00706-3

**Published:** 2022-01-21

**Authors:** Hui Wang, Cun Yu

**Affiliations:** Department of International Cooperation, Changchun Institute of Optics, Fine Mechanics and Physics, Chinese Academy of Sciences, 3888 Dong Nan Hu Road, Changchun, 130033 China

**Keywords:** Optical techniques, Optical materials and structures

## Abstract

Throughout history, there have been many outstanding women whose achievements continue to impress and amaze us today. For example, in the field of science, Madame Marie Curie was the first woman Nobel Prize winner and the only person to be awarded a Nobel Prize in two scientific fields. From China, Tu Youyou is a Nobel laureate who discovered artemisinin and dihydroartemisinin, used to treat malaria, a breakthrough in twentieth-century tropical medicine, saving millions of lives around the globe. Businesswomen such as Angela Ahrendts, a former fashion executive who helped revitalize Apple, Inc., and Sheryl Sandberg, Chief Operating Officer of Meta Platforms (formerly Facebook), are recognized as two of the world’s most influential business leaders.

Now, more than ever, women are at the forefront of developments in optics and photonics research and business. One of those leaders is Elizabeth Rogan, CEO of Optica (formerly the Optical Society and the Optical Society of America.) As the executive in charge of an organization devoted to promoting the generation, application, archiving, and dissemination of knowledge in optics and photonics worldwide, Ms. Rogan has successfully expanded the depth and breadth of Optica’s technical and global reach. Her education and expertise are in industry, finance, and strategy. She utilizes these skills in partnership with a large and technically diverse group of Ph.D. volunteers and staff specialists. Combining the efforts of these many talented people with a unity of purpose has proven to be a highly effective approach for Rogan and the association she has led for nearly two decades.

Ms. Rogan is a strong advocate for women. For instance, the association’s “Faces of Optica” campaign features a wide range of accomplished women in research and applications. And she was an enthusiastic participant in the “Rose in Science,” which celebrates the extraordinary accomplishments of women scientists.

Light Special Correspondents interviewed Elizabeth Rogan about Optica’s legacy, culture, and personal experiences as its CEO in this issue. She also discussed the reasons behind the recent rebranding of the organization and the bonds of friendship the Changchun Institute of Optics, Fine Mechanics and Physics, Chinese Academy of Sciences, and Optica have built over the years.

Please join us for an in-depth look at why this century-plus-year-old organization has a fresh new vision for the future.

## Abstract (web summary)

As the guardian and promoter of the optical business, Elizabeth Rogan will show us experience OPTICA’s profound culture, and look ahead to its missions in the new era.

## Graphical abstract

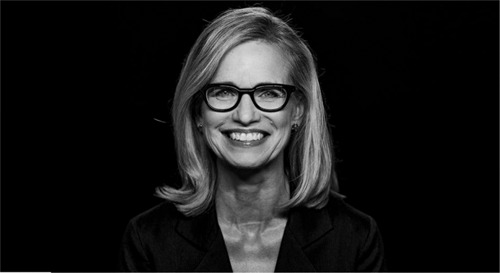

**Figure:**
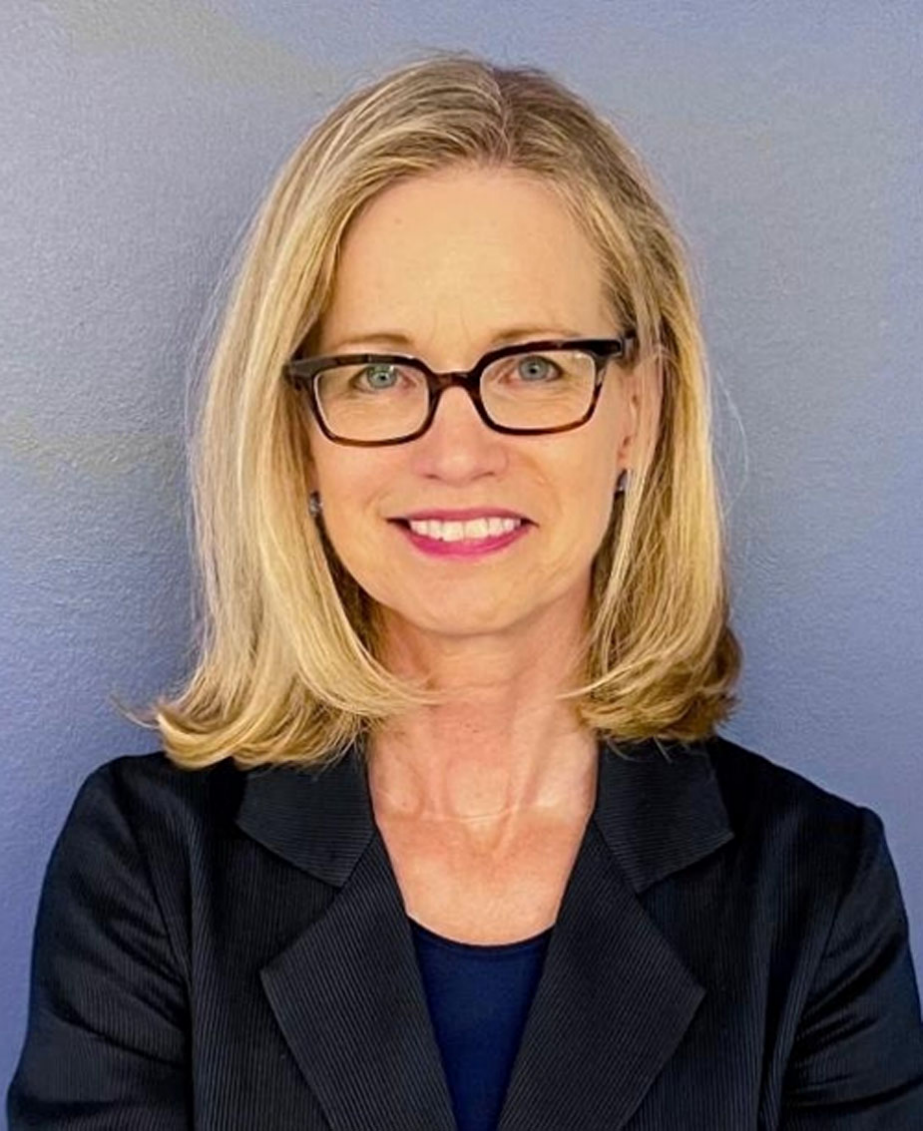

**Biography:** Elizabeth (Liz) Rogan was appointed Optica Chief Executive Officer in 2002. Previously, she served as the organization’s Chief Operating Officer.

Under Rogan’s guidance, the society’s reserves have grown to US$160M, annual budget has grown to US$50M, with 150 staff members. In 2002, she launched the Optica Foundation, a charitable entity that serves those in their student and early career phase of their professional growth. Her legacy covers the expansion of the society’s global community to include more than 432,000 scientists, engineers and business leaders from 93% of all countries.

In 2019, Rogan was named an Optica Fellow for outstanding long-term management of the Society and leadership across the optics and photonics community, guiding extensive growth in programs, member engagement, diversity, inclusivity and public policy. She was among the first class of Foreign Fellows of the Chinese Optical Society (COS).

Rogan is a CPA, an alumnus of the University of Connecticut, U.S. and a graduate of the University of Pennsylvania Wharton School of Business Executive Education Program.

**1. On September 20, 2021, Optica became the new name of the Optical Society (OSA). What’s the reason for this change?**

The change was inspired by the enormous development of the field and the substantial expansion of our association’s scope and reach. This growth motivated our leadership to carefully consider whether our name accurately reflected the Society’s work, constituents, and future. Renaming an institution that has been in existence for over 100 years is a risky and challenging task. Still, there are times when disruptive change is necessary for an organization to thrive.

After many years of discussion, the Optica rebranding effort began in 2018 when our Board of Directors launched a multi-faceted examination of our brand and its alignment with the organization as a whole. This work included wide-reaching consultation with the diverse customers we serve. Communications, branding, and design experts advised us along the way as we methodically searched for an identity that signified the vibrant, global entity we are today. We also understood that a new name must be expansive enough to remain relevant to the upcoming generations who will continue to change our field and the world for the better. At the conclusion of these studies, Optica, advancing optics and photonics worldwide, emerged as the best representation of our Society.

**2. As the society begins to use this new name, it will also launch new services and changes. Can you briefly introduce them?**

A number of our business lines were rebranded in concert with the launch of Optica. Our publishing area is now the “Optica Publishing Group,” our Corporate Membership and the Opto-Electronics Industry Development units have been consolidated and rebranded as “Optica Corporate Membership” and our charitable association has become the “Optica Foundation.” The Society as a whole, and in each of these newly titled segments, are focused on greatly expanded outreach both in terms of geography and constituent groups.

A few examples of new Optica initiatives include free meeting registration fees for members who reside in countries with low to middle-income economies and the introduction of the Optica Foundation’s substantial funded resources for students and early career professionals. The Foundation has also added a US$1M scholarship program for “Women in Optics” that will benefit 200 women scholars worldwide over the next ten years.

We are continuously adding to the events, products, and services we offer with state-of-art access options such as remote meetings, training sessions, networking resources, virtual exhibitions, and career support. This is an exciting time for the field and our organization. It is especially gratifying to know that we are all part of the community that looked ahead and re-envisioned the Society’s future to ensure it will thrive for the next hundred years.

**3. To mark the rebranding of Optica, it launched the Faces of Optica event. A total of 70 people were selected worldwide. How did you make your choices? What is the purpose of this event? What impact did it make?**

Our members are the reason Optica is a leading organization in the field. Showcasing a selection of images of these extraordinary individuals was a great way to illustrate the breadth of our community as we launched our new name and vision for the future.

Faces of Optica (viewable on www.optica.org) features a gallery of images and statements from members around the globe—including Hui Wang, Deputy Director of Department of International Cooperation, Changchun Institute of Optics, Fine Mechanics and Physics. It is a meaningful demonstration of the exceptional people who study and work in the field. Selecting those to be showcased was difficult because we serve hundreds of thousands of people in 93% of all countries, and there are so many compelling stories to tell. Award-winning photographer Sam Barker traveled the world to meet with our members and to capture their likeness in a way that relays each individual’s personality. This work was undertaken during COVID-19, and restrictions limited our ability to travel everywhere we wished to go. Despite all the challenges, we were able to achieve our goal to share the cultural and intellectual diversity of our association, and the effort has been met with much success.

**4. What competition is Optica facing now? How are you responding to those challenges?**

We are a non-profit organization focused on the promotion, generation, application, and archiving of knowledge in optics and photonics. Much of our work is directed toward the dissemination of this knowledge worldwide. Our efforts are collaborative rather than competitive. Our programs are oftentimes hosted in partnership with peer societies, universities, and commercial entities. For example, the Optical Fiber Communications Conference & Exhibit (OFC) is a joint effort with Optica, IEEE Photonics Society, and the IEEE Communications Society. Our publications portfolio includes eight journals that are co-published with other organizations---including “Chinese Optics Letters”, which is sponsored by Shanghai Institute of Optics and Fine Mechanics (SIOM), Chinese Academy of Sciences, and the Chinese Optical Society (COS). “Photonics Research” is another example that is co-published with SIOM.

One major area of competition is attracting students into our field verses other professions, like computer programming. Cultivating and enriching students studying optics and photonics is of utmost importance to Optica and the field as a whole. Our field gives young people the opportunity to make monumental contributions, and we work every day to deliver that message to young people who are selecting their area of educational concentration.

**5. Has COVID-19 affected Optica operations? What steps have you taken to deal with it?**

As was the case with organizations around the globe, the spread of COVID-19 required us to recalibrate our strategies and operations in real-time to ensure our mission continued without interruption. Thanks to the ingenuity and determination of our volunteers and staff, work carried on, and our global reach expanded. By the close of 2020, our customer base had grown just under 10% to 432,000 individuals.

By April 2020, Optica converted its meetings and conferences to virtual formats and waived all registrations fees for the remainder of the calendar year. Optica paid for its student chapters Zoom accounts so they could carry on their activities remotely—this resulted in over 2,000 student-managed sessions. There are many examples of other actions taken, such as the introduction of new publication resources and the creation of new remote programming such as the “We Are On” webinar series that is utilized by tens of thousands of viewers.

**6. Can you talk about the differences between Optica and other optical academic organizations or institutions? What is Optica’s uniqueness?**

Optica works to fulfill the mission and purpose defined by its founders in 1916. While the context has changed, the vision remains firmly in place. With this very clear direction, we are able to make progress through the collective efforts of our members and staff. Like many institutions, we devote time to strategic planning to ensure we provide services that best serve the ever-evolving needs and interests of the community and leverage our unique strengths. Our organization has always put the community first, and our profitable endeavors are reinvested back into programs that support our members and the field in the most meaningful ways possible. Every organization follows its own path. Ideally, we produce complementary resources rather than duplicate each other.

**7. Optica has set up more than 400 student chapters in universities and research institutes all over the world. Could you tell us why the society puts so much emphasis on students?**

Our university-based organizations energize and build collaborations among student members. They are a powerful force whose members contribute to the technical and social vibrancy of Optica. As a Ph.D. student, Oscar Urquidi said this about the program, “Being a part of a student chapter gives you the opportunity of projecting yourself through the lens of Optica,” he continued, “of being a real part of the Society and gaining leadership experience while you have fun in what you like the most, optics.” Urquidi highlights yet another benefit of the chapters; they are the training grounds for those who will lead the organization into the next century.

Forty-two of our chapters are based in China. Traditionally, our organization’s president travels the globe to meet with chapters. Although COVID-19 has prevented such visits, 2021 President, Connie Chang-Hasnain, who was based in China, had the opportunity to visit the Zhejiang University Student Chapter in Hangzhou. Connie said it was wonderful to connect with students after a year of isolation and fondly recalled joining the Society as a student member in 1982.

**Figure:**
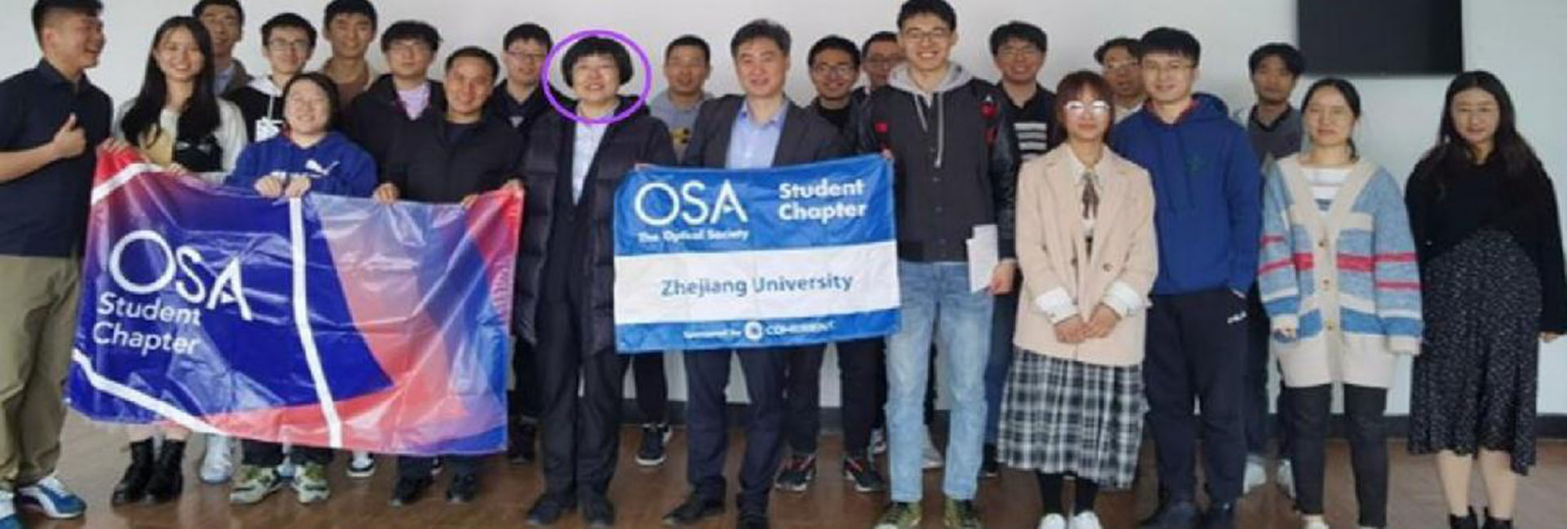
Photo of 2021 Optica President, Connie Chang-Hasnain’s visit to Zhejiang University, which took place before our name change

**8. Why do you think workers in optics should become OSA/ Optica members? What is the point of being elected OSA/ Optica Fellows?**

Optica members are part of the vibrant, global community of people with an interest in optics and photonics. Membership provides immediate access to an enormous range of benefits that can be customized around an individual’s areas of focus, such as technical topics, business issues, or career development. An Optica affiliation is a great way to make and maintain valuable contacts with colleagues around the world through online networking, volunteer activities, and in-person events.

The Fellow Member designation is highly regarded in the field, and individuals must be nominated for consideration. Those awarded Fellow Member status have served with distinction in the advancement of optics and photonics through distinguished contributions to education, research, engineering, business, and society. The number of Fellows is limited by the association’s bylaws to be no more than 10% of the total membership and the number elected each year is limited to approximately 0.5% of the current membership total.

**9. What can you tell us about Optica’s corporate culture?**

Optica’s culture is the product of our core values, unity of effort, and environment. These principles have guided our organization for a very long time. Five years ago, the Board of Directors formalized and began to better promote these values.

Sometimes referred to as “i4”, our four core values are: Innovation, Integrity, Inclusivity, and Impact. I’ll provide a recent example of each:

Innovation: Not long after the pandemic’s global spread, our Foundation and the Global Policy & Affairs Department worked with our Student Chapters to help those in need. They shared resources and technical expertise and were able to provide inexpensive optics-enabled devices to disinfect masks to those with limited protective equipment supplies.

Integrity: Providing peer-reviewed technical meeting content virtually while maintaining our high standards of integrity was a challenge we met head-on. We offered options such as live questions and answers sessions. Our meeting content was made available within 12 hours of its presentation, and every virtual session room was staffed to assure IT and logistical quality.

Inclusivity: We know that economic pressure prevents many of our peers from participating in our meetings. Our leadership decided to waive registration fees for members from low and low middle-income level countries.

Impact: Our research has and will continue to impact lives through innovation. But in addition, the scientific discovery, we seek to promote science as a means to solve some of the world’s most critical issues. One example is the Global Environmental Measurement and Monitoring Initiative. This effort engages institutions worldwide to form multidisciplinary Centers that create and share technologies and climate models. This information is then directed to assist government and industry decision-makers.

**10. Optica and Changchun Institute of Optics, Fine Mechanics and Physics (CIOMP), Chinese Academy of Sciences have long maintained a cooperative and friendly relationship. In 2011 and 2013, the two parties organized summer camp activities. In 2012, you were part of a delegation from OSA which visited CIOMP to celebrate its** **60**^**th**^ **birthday. What is your impression of CIOMP? You took part in the CIOMP’s Rose in Science event last year. How did you feel about this event?**

The 2011 and 2013 Student Camps were resounding successes on every level. Participants from around the world advanced their technical knowledge, gained new perspectives on the field, and had the opportunity to meet peers with shared interests. Everyone who traveled to the events was impressed with CIOMP’s faculty, students, and facilities. Science and engineering depend on an eco-system of collaboration, and we are delighted that the Student Camps helped to forge long-lasting professional relationships.

In 2012, we were honored to be invited to the 60^th^-Anniversary Celebration. Optica leadership took part in an outstanding program of events and festivities. It was a once-in-a-lifetime opportunity for our group, and we were gratified to have been asked to share in recognizing six decades of CIOMP excellence.

**Figure:**
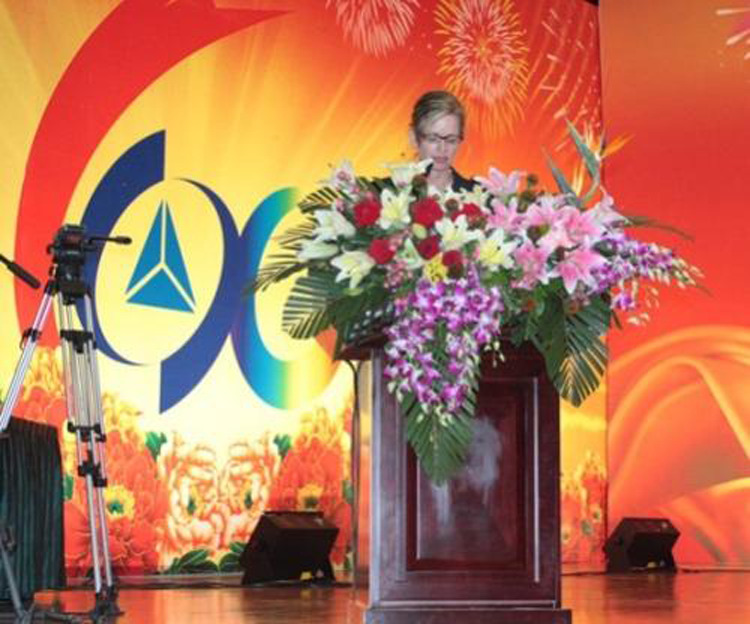
Ms. Liz Rogan gave address on the opening ceremony of CIOMP’s 60^th^ Anniversary

**Figure:**
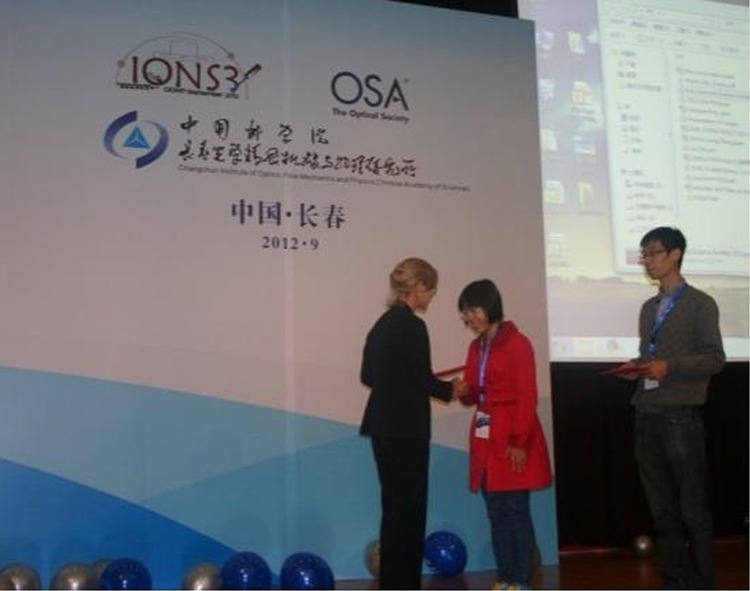
Ms. Liz Rogan awarded prizes to students in IONS jointly held by OSA and CIOMP

**Figure:**
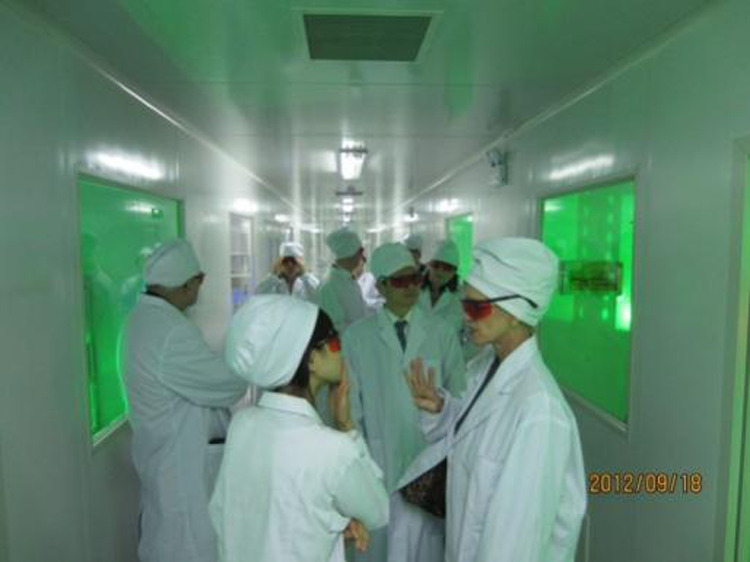
The OSA delegation visits the Changchun New Industries Optoelectronics Technology Co., Ltd. based at CIOMP

**11. You graduated from the Wharton School of the University of Pennsylvania, which is widely considered the best business school in the world. So why did you choose to work for the Optical Society (OSA), now renamed Optica?**

I graduated from a special Executive Leadership Program at the Wharton School. It was an outstanding educational program tailored to professionals who hold senior management positions. Directing my knowledge, experience, and energy to Optica was a choice that was made easily, thanks to the extraordinary people in the optics and photonics community. The impact of their work and the collegiality of the field impressed me and made me want to devote my career to the organization. It proved to be an excellent choice.

**12. What other jobs did you have before this one at Optica? How did they affect you?**

I was fortunate to have had a wide range of professional experiences in politics, performing arts, banking, and public accounting sectors prior to joining Optica nearly 30 years ago. Each of these positions was a learning experience that helped me to develop and grow as a leader and to prepare me for my role as a CEO.

**13. Have you had any great opportunities while working at Optica? What did you do at that time? What have you gained from working at Optica for so many years?**

My time with Optica has provided too many great opportunities to list. I am truly grateful for these experiences. One example was traveling to Stockholm, Sweden to attend the Nobel Prize ceremonies as a guest of Optica 2013 President Donna Strickland when she was recognized in 2018. Another example was being included in the CIOMP 60^th^-anniversary celebrations. These two examples of high points made lasting impressions on my personal and professional perspective.

**14. Have you ever run into difficulties at work? Can you share with us some incidents that you remember most clearly?**

Being a CEO is rewarding and challenging. Leading Optica from the onset of COVID-19 through today is an example of why it is critical for executives to have a broad range of skills from which to draw. The pandemic affected every facet of our organization. Having an understanding of our entire operation—from financial and operations to member/student services, IT and virtual delivery options, helped me and our Board of Directors to make sound decisions that will impact the organization and the community for many years to come.

**15. What is the biggest turning point in your career?**

Transitioning from the role of Chief Operating Officer (COO) to Chief Executive Officer (CEO) was the biggest turning point in my career. At the most fundamental level, the role of COO is an internally-focused position, one that concentrates on optimizing an organization from within. By contrast, a CEO must lead in context and in collaboration with the external environment.

**16. As the CEO of Optica, what can you tell us about your understanding of leadership?**

I have had the opportunity and honor to work directly with more than 20 Optica presidents. Each one of these accomplished individuals is a role model in areas that include leadership, critical thinking, communications, community service, and so much more. Working with our presidents and board members is one of the most gratifying and educational parts of my role as CEO.

**17. Do you think there is a difference in leadership styles between men and women?**

In my experience, professional affiliation differentiates leadership style more so than gender. Academia, government, and the corporate sectors operate very differently; as a consequence, their senior managers often approach leadership and decision-making in ways reflective of their institutions. This is an asset to Optica, and we are glad to draw our volunteer leaders from all spheres.

**18. As far as we know, you are probably one of the few serving CEOs of international academic organization who does not have a scientific research background. What do you think is the reason for this amazing achievement, from the perspectives of Optica and yourself?**

My expertise supports the financial health and strategic growth of Optica. When I joined the Society, the association was half the size it is today with limited engagement outside the U.S. Since then, our programs, products, and services have grown significantly. Our expansion is achieved through the partnership of technical experts and professional staff in areas that include publications, corporate and individual membership, conferences, awards, and special outreach programs. This approach has proven to be an excellent model for running our vibrant and growing organization.

**19. Have there been any people without scientific research background selected as committee members or fellow of Optica? Why?**

All of our Fellows have made noteworthy achievements in science and technology. While most of our council and committee members are engaged in science, we call upon people who are directly engaged in research and development. For example, librarians, conference exhibitors and the CEOs of partner companies will provide us with advice and support on ways our products and services can best meet their needs.

**20. We know that Optica has always adopted a democratic voting system. As the CEO, how do you communicate with scientists, get them to accept your ideas and give strong support to you?**

Ideas are most often cultivated by our community and carefully discussed with a broad range of stakeholders. This is an excellent way to ensure diversity of thought and acceptance. As CEO, I serve as a trusted, transparent source for the Board members who are the association’s decision-makers.

OSA is a “member-driven” society which is reflected through its governance structure. Responsibilities are allocated to topic-specific councils comprised of nominated volunteers. These bodies include standing and ad hoc committees where necessary. Many councils include an Optica Board member who regularly provides the Board of Directors (BoD) with status reports and recommendations for consideration and feedback. To ensure unity of effort, councils incorporate relevant segments of the Strategic Planning Council Long-term Plan into their multi-year goal-setting and focus areas. Every governing body has a designated Optica staff member to provide professional-level support.

**21. When you interview new employees, what qualities do you look for?**

Optica employees include people with specialized skills in areas such as IT, events management, governance, philanthropy, publishing, and much more. While each staff member contributes in a unique way, there are qualities everyone shares. They include a dedication to providing excellent products and services, tenacity, creativity, resourcefulness, flexibility, honesty, and a sense of optimism. I look for these same traits when I interview new prospective employees.

**22. What was your dream when you were a kid? Has it come true?**

When I was a child growing up on the east coast of the United States, I dreamed of seeing the countries of the world and meeting interesting people who lived there. Happily, that dream has come true.

**23. In your career, has anyone had a major influence on you? In what way?**

Literally, every Optica president has been a major professional and personal influence. The past, present, and upcoming presidents comprise the “Presidential Chain”, a four-year procession that lends continuity to OSA’s most senior leadership body and provides a multi-year opportunity to learn from these extraordinary individuals.

**24. How do you see the role of women in the development of optics?**

I am very optimistic and enthusiastic about the growing presence of women in the optics and photonics field. Diversity accelerates innovation, and the intellect and creativity of the women in science drives discoveries that will improve life as we know it.

**25. What advice and suggestions would you give our young audience on life and career?**

My advice is to fully engage in the areas that interest and inspire you. Also, do not be afraid of failure. We often learn much more from “mistakes” than successes. Learning why something didn’t work is an opportunity to grow and strengthen your resolve. And, as you pursue a profession in the science of light, please know that Optica stands ready to serve you throughout your career.

**Light special correspondents**

*Hui Wang is the Deputy Director of the Office of International Cooperation in the Changchun Institute of Optics, Fine Mechanics and Physics (CIOMP), Chinese Academy of Sciences (CAS). She currently works on international communication and cooperation for the CIOMP and was a founding member for the Nature Publishing Group and CIOMP joint journal Light: Science & Applications. She is the founder of “Rose in Science” and has published several articles in Acta Editologica, International Talent, Light: Science & Applications, etc., and was invited to take an interview by SPIE Women in Optics, which was published in 2015.*

**Figure:**
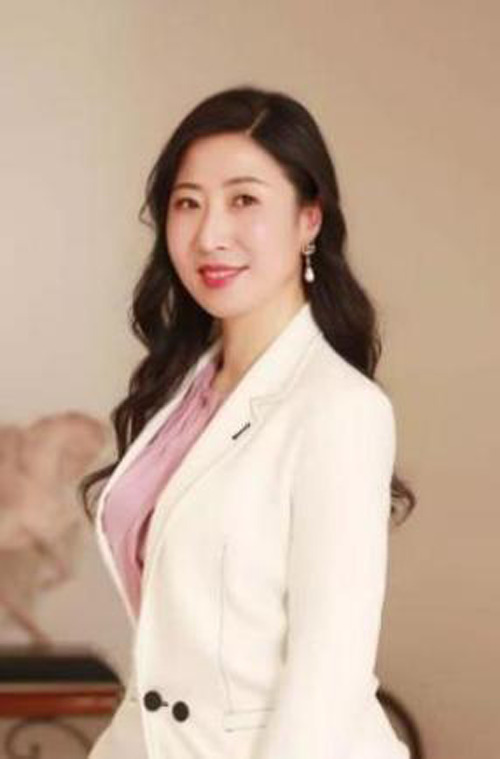

*Cun Yu works at the Office of International Cooperation in the Changchun Institute of Optics, Fine Mechanics and Physics (CIOMP), Chinese Academy of Sciences (CAS). Her main duties cover the Sino-Belarus International Innovation Center, international cooperation projects and exchanges between CIOMP and institutions in Belarus, Ukraine and Russia. She has published multiple articles in the journal International Talent, Light: Science & Applications, and is a member of CSA’s Science & Technology Translations Association.*

**Figure:**